# Effects of Rare Coding Variants in Severe Early-Onset Obesity Genes in the Population-Based UK Biobank Study

**DOI:** 10.1210/clinem/dgaf132

**Published:** 2025-02-28

**Authors:** Raina Y Jia, Sam Lockhart, Brian Y H Lam, Yajie Zhao, Katherine A Kentistou, Eugene J Gardner, I Sadaf Farooqi, Stephen O’Rahilly, Felix R Day, Ken K Ong, John R B Perry

**Affiliations:** University of Cambridge, Medical Research Council Epidemiology Unit, Institute of Metabolic Science, Cambridge CB2 0SL, UK; University of Cambridge, Medical Research Council Metabolic Diseases Unit, Institute of Metabolic Science-Metabolic Research Laboratories, Cambridge CB2 0QQ, UK; Queen's University Belfast, School of Medicine, Dentistry and Biomedical Sciences, Wellcome-Wolfson Institute for Experimental Medicine, Belfast BT9 7BL, UK; University of Cambridge, Medical Research Council Metabolic Diseases Unit, Institute of Metabolic Science-Metabolic Research Laboratories, Cambridge CB2 0QQ, UK; University of Cambridge, Medical Research Council Epidemiology Unit, Institute of Metabolic Science, Cambridge CB2 0SL, UK; University of Cambridge, Medical Research Council Epidemiology Unit, Institute of Metabolic Science, Cambridge CB2 0SL, UK; University of Cambridge, Medical Research Council Epidemiology Unit, Institute of Metabolic Science, Cambridge CB2 0SL, UK; University of Cambridge, Medical Research Council Metabolic Diseases Unit, Institute of Metabolic Science-Metabolic Research Laboratories, Cambridge CB2 0QQ, UK; University of Cambridge, Medical Research Council Metabolic Diseases Unit, Institute of Metabolic Science-Metabolic Research Laboratories, Cambridge CB2 0QQ, UK; University of Cambridge, Medical Research Council Epidemiology Unit, Institute of Metabolic Science, Cambridge CB2 0SL, UK; University of Cambridge, Medical Research Council Epidemiology Unit, Institute of Metabolic Science, Cambridge CB2 0SL, UK; University of Cambridge, Department of Paediatrics, Cambridge CB2 3RF, UK; University of Cambridge, Medical Research Council Epidemiology Unit, Institute of Metabolic Science, Cambridge CB2 0SL, UK; University of Cambridge, Medical Research Council Metabolic Diseases Unit, Institute of Metabolic Science-Metabolic Research Laboratories, Cambridge CB2 0QQ, UK

**Keywords:** obesity, variant classifications, population genetics, whole-exome sequencing

## Abstract

**Context:**

Clinical case–based studies have identified rare pathogenic variants in several genes as causes of severe early-onset obesity, but their penetrance and interaction with polygenic susceptibility in the general population remain unclear.

**Objective:**

We analyzed the United Kingdom Biobank (UKBB) whole-exome sequence data to assess the effects of heterozygous variants in 9 previously reported genes on adult body mass index (BMI) and recalled childhood adiposity.

**Methods:**

Among 419 581 UKBB participants, we identified heterozygous carriers of coding variants that were (1) experimentally characterized as loss of function (LoF), or (2) bioinformatically predicted as rare (minor allele frequency <0.1%) LoF. We assessed variant-level and gene-level population penetrance of obesity and associations with adult BMI and recalled childhood adiposity, and tested the statistical interaction between rare variant carriage and a BMI polygenic score.

**Results:**

Considering experimentally characterized LoF variants (excluding *MC4R*), we identified 22 heterozygous and 2 homozygous variants in 3 autosomal recessive genes (*POMC*, *PCSK1*, *LEPR*), and 3 autosomal dominant genes (*SH2B1*, *SIM1*, *KSR2*) with at least 10 carriers in the UKBB. Obesity penetrance among carriers ranged from 8% to 29% (median 23%), and none was significantly different from noncarriers (24%, all *P* > .05). For bioinformatically predicted rare LoF variants, gene-based burden tests showed that carriage of heterozygous variants in *MC4R*, *PCSK1*, and *POMC* was associated with higher adult BMI (effect sizes ranged from 0.5 to 2.5 kg/m^2^, all *P* < .003), with no significant interaction effects with common variant polygenic risk of BMI.

**Conclusion:**

This study provides the population-specific report of variant penetrance of known obesity genes and confirmed the heterozygous rare variant effects in *MC4R*, *POMC*, and *PCSK1*. We also underscore the utility of population-based studies in supporting variant classifications.

Obesity is clinically defined in adults as a body mass index (BMI) over 30 kg/m^2^ and in children is based on sex- and age-specific BMI percentiles (1). Most cases of childhood and adult obesity are polygenic, influenced by multiple genetic and environmental factors (2, 3). However, there are also rare monogenic forms of obesity, characterized by early-onset obesity (typically before age 2) with hyperphagia (nonsyndromic obesity), or early-onset obesity accompanied by other congenital neurodevelopmental abnormalities (syndromic obesity) (4). Studies of patients with severe early-onset obesity have identified several genes in index patients involved in the leptin–melanocortin pathway (5) in which high penetrant variants significantly disrupt the function of hypothalamic satiety signaling and appetite regulation and lead to excessive food intake (6). For instance, carriage of homozygous variants in *LEP* (7), *LEPR* (8), *POMC* (9), and *PCSK1* (10) and compound heterozygous variants in *POMC* (9), *PCSK1* (11), and *LEPR* (12) have been reported to cause severe early-onset obesity with high penetrance. Variable effects (ranging from no effect to mild effect to moderate effect) have been reported in heterozygous carriers for *POMC* (13), *PCSK1* (14-16), and *LEPR* (8, 12) on childhood obesity and for *LEP* (12) on increased fat mass. The effect of these genes on the lifelong susceptibility to severe obesity in the general population remains unclear (17-19). *MC4R*, on the other hand, has been reported to be codominant with a variable penetrance in early-onset and childhood obesity (20) as well as in the susceptibility to obesity in population-based studies (21, 22). The frequency of *MC4R* loss of function (LoF) variants among children with obesity is subject to the ways study participants are selected. For instance, in a cohort of ∼2000 children (under 18) with obesity, those with a family history of obesity had a higher frequency of *MC4R* LoF variants (2.31%) than in sporadic cases (1.08%) (23).

With advancements in DNA sequencing in the 2000s, several other genes have also been discovered in patients with severe early-onset obesity. Many of which were discovered in patients with syndromic obesity. For instance, carriage of a deletion containing the *SH2B1* region (24) or carriage of heterozygous variants in *SH2B1* (25), *SIM1* (26-29), *NTRK2* (30, 31), and *KSR2* (32) have all been reported in severe early-onset obesity. Some of these cases were accompanied by neurodevelopmental and/or behavioral phenotypes, and, where pedigrees were available, most cases showed incomplete penetrance. These genes have been included for screening of pathogenic variants in early-onset obesity in case–control or case report studies up until the recent 5 years (15, 33-38). Whether rare, heterozygous variants on these genes also contribute to the susceptibility of lifelong obesity outside early-onset obesity cases remains uncertain (17-19).

The motivation of this study was 2-fold. First, more evidence is needed on the effects of rare variants in genes listed on clinical genetic panels, as clinical testing for childhood obesity often identifies “variants of uncertain significance” (39). Second, there is a growing interest in understanding the heterozygous effects of rare variants in known obesity genes in the broader population (19, 40), as this may help to identify individuals who might benefit from early therapeutic interventions (41). To address these gaps, we leveraged whole-exome sequence data from ∼400 000 individuals of European ancestry in the United Kingdom Biobank (UKBB) study (42) to assess the effect of rare protein-coding variants in these genes on the risk of obesity. While case-based study designs are suited for identifying variants of large effect in clinically ascertained patients, the few cases often made statistical testing unfeasible.

Here, we first sought to identify individuals carrying previously reported in vitro characterized LoF variants and examined their obesity penetrance and association with BMI in the context of a population using UKBB. We next examined the gene-level associations of bioinformatically predicted rare LoF variants with BMI and self-reported childhood adiposity. Consistent with previous observations for *MC4R*, we find incomplete penetrance of obesity for the genes examined using predicted LoF variants in the general population. Additionally, this study highlights the utility of population-based studies in filtering out clinically identified variants that lack association with surrogate phenotypes.

## Materials and Methods

### Exome Sequencing Quality Control

The UKBB study has provided whole-exome sequencing for the majority of the study participants (43, 44). We included variants that passed the quality control pipeline previously described in Gardner et al (45). Briefly, based on the VCF files with variants mapped to the GRCh38 reference using BWA-MEM and DeepVariants (42), we split and left-corrected multi-allelic variants into separate alleles using “bcftools norm” (46). Next, we used “bcftools filter” to include variants that fulfilled the following criteria: (1) read coverage ≥7; (2) genotype quality score ≥20; (3) those that did not exhibit strand bias—this was tested by a binomial test for an expected contribution of 50% for the alternative allele—alternative alleles with *P* binomial ≥ 1×10^−3^ were included; and (4) those where the proportion of individuals with missing genotype was less than 50%.

### Variant Annotations

We used ENSEMBL (47) Variant Effect Predictor v104 (48) to annotate the variants. Each protein-coding variant was annotated based on either the “MANE SELECT” or the “CANONICAL” transcript, where the transcript with a more severe predicted consequence was selected, as described previously (45). We then used Variant Effect Predictor plugins to define variants of interest based on their predicted functional impact. Briefly, for protein-truncating variants (PTVs) we included LOFTEE classified “high confidence” PTVs in the analysis; this classification was based on an algorithm that further filtered stop-gained, splice-site, or frameshift variants based on a number of criteria, as detailed in Karczewski et al (49). For defining likely damaging missense variants, we used CADD (47, 50) and REVEL (51) scores. Both are tools that combine multiple algorithms that encompass various aspects of predictive features, such as sequence homology, conservation scores, and the physical–chemical similarities between amino acids. While CADD was trained to distinguish simulated variants from all evolutionary conserved human variants across the genome, REVEL was trained to distinguish recently discovered rare pathogenic variants from neutral missense variants based on curated databases. In summary, we defined 2 classes of variants for analysis: (1) LOFTEE-defined high-confidence PTVs (HC-PTVs); and (2) damaging missense (DMG-MISS) variants based on the stringent definition that a variant has both CADD score ≥25 and REVEL score ≥0.7.

### Study Population and Phenotypes

Adult BMI values (UKBB data field 21001) and comparative body size at age 10 (UKBB data field 1687) were extracted from the UKBB research analysis platform (https://ukbiobank.dnanexus.com/). We included 419 581 individuals in the UKBB of European ancestry with BMI measurements and 414 023 with self-reported childhood body size. We reported the primary association test results for predicted LoF variants using raw BMI values. To account for potential outliers as the BMI measurement in the UKBB has a modest right skew, we repeated all tests using the rank-based inverse normal transformed BMI and confirmed consistent effect estimate and statistical test results. Self-reported size as child at age 10 (UKBB data-field 1687) asked participants to describe their body size relative to their peers when they were 10 years old as “thinner,” “plumper,” or “about average.” We treated it as a continuous variable, with −1 representing thinner, 0 representing average, and 1 representing plumper. While this recalled childhood body size is a proxy measure of childhood adiposity, we previously confirmed that it has a strong genetic correlation with childhood BMI (*R*_g_ = 0.94) (52).

Our gene burden tests of rare variants modeled the presence or absence of variants of interest in a gene as an indicator variable. This was regressed against the outcome trait in a mixed linear regression model using BOLT-LMM v2.3.6 (53). The models for BMI and comparative childhood size were adjusted for sex (UKBB data field 22011), age at assessment (UKBB Data-field 21003), age^2^, and the first 10 genetic principal components as calculated in Bycroft et al (42) (UKBB data field 22009.1-10).

### Construction of BMI Polygenic Risk Scores

A genome-wide polygenic score (PGS) for adult BMI was constructed for 419 581 individuals in UKBB of white European ancestry who had genotype array, exome sequence, and BMI data. To construct the BMI PGS, we used BMI genome-wide association study summary statistics reported by Locke et al (54), which did not include samples from the UKBB (available from: https://portals.broadinstitute.org/collaboration/giant/index.php/). These data included 2 113 400 SNPs from 322 154 participants of European ancestry. For the genotype data on UKBB participants, single nucleotide polymorphisms (SNPs) were removed if they had a minor allele frequency (MAF) <0.1%, Hardy Weinberg equilibrium *P* < 1×10^−6^, or genotype missingness >10%. Additionally, SNPs that were mismatched between the 2 data sets were excluded.

We assessed 4 methods to construct the PGS: lassosum v0.4.5 (55), PRSice v2.3.5 (56), PRS-CS (latest update in 2021) (57), and Plink v1.9.0 (58). We compared the proportion of variance (*R*^2^) explained for adult BMI in models containing the PGS and covariates (sex, age and age^2^, and the first 10 ancestral principal components). The PGS generated by lassosum (using the default parameter settings) provided the highest *R*^2^ (11%) and was used in subsequent analyses. Lassosum is a penalized regression method that accounts for linkage disequilibrium using a reference panel, which we constructed based on a random subsample of 25 000 unrelated European UKBB participants.

### Interaction Effects Between Polygenic Risk Scores and Rare Variant Carrier Status

To examine the interaction between rare variant carrier status and the BMI PGS, we included an interaction term between the PGS and a binary variable representing rare variant carrier status in linear regression models. We also investigated interactions between rare variant carrier status and individual common variants in UKBB genome-wide association study array data using the “epistasis” function in Plink v1.9 (58). Multiple testing correction was applied based on 821 BMI independent signals (*P* < 6×10^−5^).

## Results

### Population-Based Penetrance of Adult Obesity of Experimentally Characterized LoF Variants in Obesity Genes

We conducted a literature review for 8 of the 9 monogenic obesity genes (*POMC*, *PCSK1*, *LEPR*, *LEP*, *SH2B1*, *NTRK2*, *SIM1*, and *KSR2*) to identify variants that have been implicitly or explicitly reported as pathogenic in case–control studies of children or adults with severe obesity, or case reports of children with severe early-onset obesity (Fig. S1 (59) and Table S1 (59)). We omitted *MC4R* as a similar population-based analysis has been previously reported (21, 60, 61). We included articles published between 2005 and 2023 (to cover the era of high-throughput DNA sequencing). We extracted variants identified in patients that were confirmed with either Sanger sequencing or next generation sequencing and were experimentally characterized as complete or partial LoF (at least 1 line of experimental evidence suggesting LoF).

**Figure 1.**
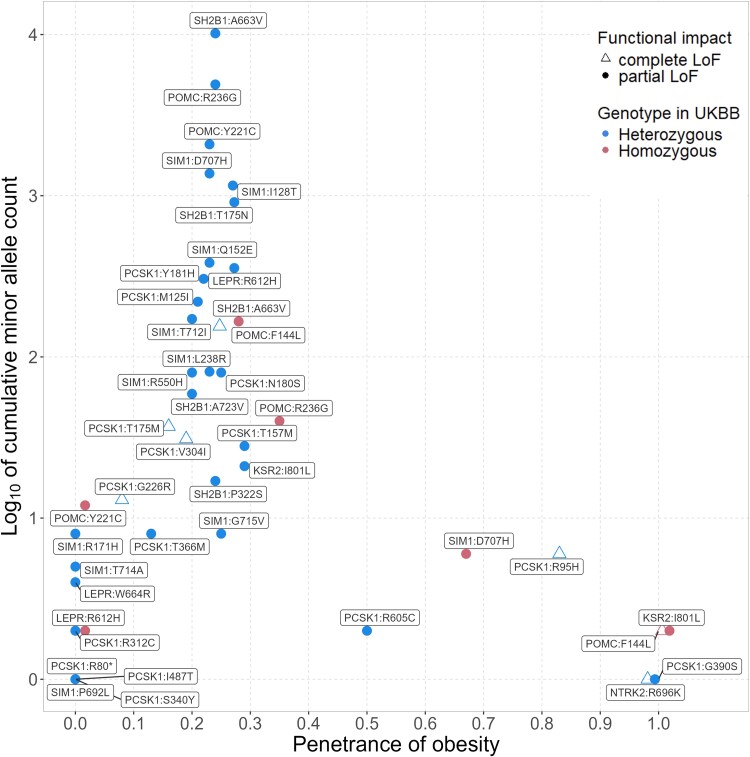
Penetrance of obesity in the UKBB among carriers of reported functionally characterized variants. The cumulative minor allele count is the sum of minor alleles in the UKBB. Further details of the variants identified from literature are available in Table S2 (59).

We identified 82 missense variants and 14 PTVs from 22 studies (Fig. S1 (59), Table S2 (59)). Of these, 36 missense variants and 1 PTV from 14 studies were present in the UKBB, in 7 of the 8 genes examined (*POMC*, *PCSK1*, *LEPR*, *SH2B1*, *NTRK2*, *SIM1*, and *KSR2*). Most of the identified variants were rare (MAF < 0.1%) in UKBB except for *SH2B1*:A663V (MAF = 1.2%; Fig. 1; Table S2 (59)). Twenty-six (74%) of these 35 missense variants were predicted by at least 1 in silico tool as LoF with high confidence (CADD ≥ 25 or REVEL ≥ 0.5). We identified 21 variants with least 10 carriers in the UKBB (Fig. 1; Table S2 (59)). Among these, 11 heterozygous and 1 homozygous variant were found in 3 reportedly autosomal recessive genes (*POMC*, *PCSK1*, and *LEPR*) associated with severe early-onset obesity. The obesity penetrance (the proportion of variant carriers with BMI >30 kg/m^2^) ranged from 20% to 28% (median = 23%; overall obesity penetrance in the UKBB = 24%). Additionally, another 11 heterozygous and 1 homozygous variant were identified in 3 reportedly autosomal dominant genes (*SH2B1*, *SIM1*, and *KSR2*), with an obesity penetrance ranged from 8% to 35% (median = 24%; overall obesity penetrance in the UKBB = 24%).

### Population-Based Penetrance of Adult Obesity Using Predicted LoF Variants Collapsed by Genes

We next assessed the gene-level obesity penetrance of bioinformatically predicted rare LoF variants in the 9 genes, assuming rare predicted LoF variants have a consistent direction of effect on the trait. By grouping these variants, we aimed to provide a population-level estimate of the penetrance of likely LoF, rare variants in putative monogenic obesity genes. This is complementary to the association test with BMI described below. Notably, this estimate is subject to potential inaccuracies in variant annotation and functional prediction. It also differs from the variant-level penetrance analysis reported earlier, which focused on variants with prior experimental evidence of functional impact and suspected pathogenicity in early-onset obesity.

We defined 2 variant classes, high-confidence protein-truncating variants (HC-PTVs) and DMG-MISS variants, using the overlap of 2 variant consequence predictive tools with stringent thresholds (CADD > 25 and REVEL > 0.7; “Materials and Methods”). The gene-level obesity penetrance among carriers (the proportion of carriers with at least 1 qualified variant in a specific gene who had a BMI > 30 kg/m^2^) ranged from 22% to 41% for HC-PTVs and from 13% to 36% for DMG-MISS variants across the 9 genes (Table S3 (59)). Compared to the 24% population prevalence of obesity among noncarriers (not carrying a qualifying variant in any of the 9 genes examined), we observed a higher obesity penetrance in carriers of *MC4R* HC-PTVs (2-sided proportional z-test: *P* = 5×10^−8^), *PCSK1* HC-PTVs (*P* = 2×10^−4^), *POMC* HC-PTVs (*P* = .01), *MC4R* DMG-MISS (*P* = 9×10^−8^), and *POMC* DMG-MISS (*P* = 8×10^−4^) (Table S3 (59)).

For *MC4R*, the prevalence of carriers of rare bioinformatically predicted DMG-MISS and HC-PTVs combined in the UKBB was 1 in 1000 (Table S3 (59)). This is lower than the previously reported prevalence of 3 in 1000 for experimentally characterized LoF variants (including missense and PTVs) in a UK birth cohort of ∼6000 European participants (21), and lower than the prevalence reported in clinically ascertained patients with severe early-onset obesity (∼10-50 per 1000 in samples with ∼500-2300 patients) (20, 23, 62). The prevalence of carriers of a predicted LoF variant in the other genes in the UKBB ranged from 0.02 per 1000 (*NTRK2* HC-PTV) to 2.35 per 1000 (*PCSK1* DMG-MISS) (Table S3 (59)).

Among the 9 genes, we identified only 2 homozygous carriers of a predicted LoF variant in the UKBB. One individual was homozygous for a DMG-MISS variant in *KSR2* (NM_173598.6.2401A>C, p.Ile801Leu; CADD = 28 and REVEL = 0.7). This individual was obese as an adult (BMI = 33 kg/m^2^), self-reported as “plumper than average” during childhood and had an average BMI PGS (50th percentile). Heterozygous carriage of this variant has previously been reported as a suspected penetrant cause of severe early-onset obesity (32). Previous in vitro data characterized this variant as a partial LoF with an effect on glucose oxidation, but not fatty acid oxidation (32). In the UKBB, we identified 21 heterozygous carriers with a mean BMI of 27.8 kg/m^2^ (range 21.5-36.8 kg/m^2^) and an obesity penetrance of 29% among carriers (*P* = .7 compared to noncarriers).

The second individual was homozygous for a DMG-MISS variant in *POMC* (NM_000939.4:c.430T>C, p.Phe144Leu; CADD = 32 and REVEL = 0.89). This individual was an adult with obesity (BMI = 31 kg/m^2^), who reported an “average body size at age 10” and had an above-average BMI PGS (86th percentile). Heterozygous carriage of this variant has previously been associated with early-onset obesity and shown to abolish the binding of α-MSH to MC4R in vitro (13). We identified 154 heterozygous carriers of this variant in the UKBB, with a mean BMI of 28.3 kg/m^2^ (range 18.7-53.5 kg/m^2^) and obesity penetrance of 27% (*P* = .7 compared to noncarriers). Thus, this large-scale population-based analysis suggests that homozygous carriage of either of these 2 variants is unlikely to be clinically relevant.

### Effects of Rare Variant Gene Burden on Adult BMI and Childhood Body Size

**Figure 2.**
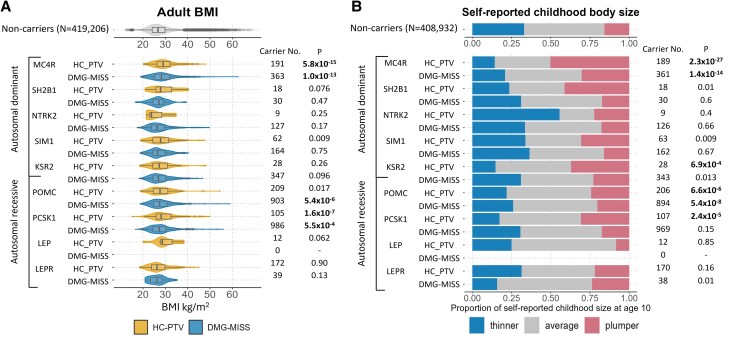
Main effects of predicted rare loss of function variants in reported monogenic genes on (A) adult BMI and (B) comparative childhood body size. Noncarriers are individuals who do not carry a variant of interest in any of the 9 genes examined. Childhood body size at age 10 was based on 3 response options to the self-reported question: “leaner,” “average,” or “plumper” at age 10. *P* < .0003 (corrected for 8 genes × 2 variant masks) are highlighted in bold. HC_PTV, high-confidence protein-truncating variants; DMG-MISS, damaging missense variants (defined as the overlap of CADD > 25 and REVEL > 0.7, “Materials and Methods”). Full gene-based burden test results are available in Table S2 (59).

We next extended our analyses to assess the impact of the predicted LoF variants on adult BMI and a comparative measure of self-reported childhood body size at age 10 (“Materials and Methods”). We identified in the UKBB 3742 carriers of at least 1 variant of interest across the 9 genes with adult BMI data, and 3707 carriers with self-reported childhood body size data. Carriers of HC-PTVs in *MC4R* (n = 191), *PCSK1* (n = 105), and carriers of DMG-MISS in *MC4R* (n = 363) and *POMC* (n = 903) exhibited both a higher adult BMI (Fig. 2A; Table S4 (59)), and a plumper than average childhood body size (Fig. 2B; Table S4 (59)) compared with noncarriers. Additionally, carriers of DMG-MISS variants in *PCSK1* (n = 986) showed a higher adult BMI (Table S4 (59)), but no significant effect on childhood body size (Fig. 2B). Finally, carriers of HC-PTVs in *POMC* (n = 206) and *KSR2* (n = 28) had a plumper than average childhood body size (Table S4 (59)) but no significant effect on adult BMI (Fig. 2A).

Furthermore, we investigated potential protein domain–specific effects in NTRK2, as previous evidence indicated that only rare variants located at the active site of the tyrosine kinase domain in *NTRK2* were pathogenic and associated with syndromic obesity (30, 36). Among the 8 previously experimentally characterized heterozygous LoF variants (30, 36), only 1 variant (R696K) was identified in the UKBB, found in a single carrier who was severely obese (BMI = 41 kg/m^2^) but reported an average body size at age 10. We further examined the combined effect of heterozygous predicted DMG-MISS variants located in the tyrosine kinase domain of *NTRK2* (41 out of 53 DMG-MISS variants found in *NTRK2*) but found no significant association with adult BMI (carrier n = 93, *P* = .36) or self-reported childhood body size at age 10 (carrier n = 91, *P* = .92) (Fig. S2 (59)). Experimental data indicated that the R696K variant impacts arginine residues at the active site of the kinase domain. Our findings of null associations using predicted DMG-MISS variants in the tyrosine kinase domain suggest that focusing on this domain may have limited predictive value for identifying true pathogenic variants in *NTRK2*.

### Interaction of Predicted LoF Variants in Obesity Genes and BMI Polygenic Score

Previous studies have suggested that pathogenic *MC4R* variants may modify the effects of common genetic variants associated with BMI (60). We examined whether the impact of carrying predicted rare LoF variants in each of the 9 genes interacts with common variant BMI PGSs (“Materials and Methods”). A nominally significant positive interaction effect was observed between BMI PGS and carriage of damaging missense (DMG-MISS) variants in *MC4R* with respect to childhood body size at age 10 (β = .07, SE = 0.04, *P* = .049; Table S5 (59)). This indicates that the presence of both a higher PGS of BMI and the carriage of rare *MC4R* missense LoF variants may enhance (with a small additional effect on top of the sum of the effect from rare variants and PGS, respectively) the risk of childhood susceptibility to higher adiposity (body size). However, no significant interaction effects were found at the multiple testing corrected threshold of *P* < .005 (adjusted for 9 tests per trait) (Table S5 (59)). No additional significant associations between gene-variant groups and adult BMI or childhood body size were identified in the interaction models compared to the results presented in Fig. 2.

Moreover, we did not find any multiple testing corrected significant interaction effects between individual common variant signals for adult BMI and the carriage of rare predicted LoF variants across the 9 genes. These results suggest that rare variants in these genes generally act independently and additively with BMI associated common variants and BMI PGS (the effect of both variables on the trait is the sum of the individual effects, and the presence of one does not affect the effect of the other) to influence the susceptibility of adult and childhood adiposity.

## Discussion

We focused on 9 genes that are commonly included on clinical genetic testing panels for severe early-onset obesity, such as the UK NHS genomic panel (https://panelapp.genomicsengland.co.uk/) and the US National Institutes of Health Genetic Testing Registry (63)(http://www.ncbi.nlm.nih.gov/gtr/). While we might expect the biggest impact of monogenic obesity genes to be in childhood, we were limited by the lack of genomic sequence data of sufficient scale in that age group. Here our study provides informative insights into whether these monogenic obesity genes also contribute to the lifelong susceptibility to obesity. This study also aligns with the ongoing calls for cautious interpretation and reporting of variant-level disease penetrance (64, 65). Various factors can influence study estimates of penetrance, including ascertainment bias in the study design and biological complexities, such as variable expressivity in the genotype–phenotype associations.

In this study, we reassessed the penetrance of previously reported, suspected pathogenic variants for early-onset obesity as well as rare predicted LoF variants in the target genes. Our observations recapitulated previous findings regarding the obesity-associated *MC4R* gene (21) and the type 2 diabetes–associated *HNF1A* gene (66), that the penetrance of predicted LoF variants in known genes in clinically unselected cohorts tends to be lower than originally reported in studies of rare cases. Moreover, in line with the current efforts to systematically classify clinical variant, such as the ACMG/AMP (American College of Medical Genetics and Genomics and Association of Molecular Pathology) guidelines (67-69), and the development of curated database, such as ClinGen (70), this study demonstrated the utility of population-based studies as one line of evidence for variant classifications. While high-frequency variants in the population are likely benign, variants seen in rare clinical disorders but absent from population biobanks warrant further assessment.

By analyzing the UKBB data, we confirmed evidence of haploinsufficiency in *MC4R*, *PCSK1*, and *POMC* genes underlying the risk of common obesity, where carriers with predicted LoF variants showed a 0.5 to 2.5 kg/m^2^ increase in BMI than noncarriers. Early index cases of rare monogenic obesity were reported in patients with compound heterozygous variants in *PCSK1* (11) and homozygous variants in *POMC* (9). In this study, using predicted PTVs (assumed to result in complete LoF) and DMG-MISS variants (predicted to affect protein function to some degree), we found strong evidence for a haploinsufficiency effect in *POMC* on the predisposition to increased adiposity in both adulthood and childhood. This converges with the previous pedigree-based studies of *POMC*, which suggested that while heterozygous carriage of experimentally characterized partial LoF variants does not contribute to monogenic obesity, it might still underly the susceptibility to increased adiposity (18, 71).

In contrast, a previous case–control study in adults found that only experimentally characterized nonsense heterozygous variants in *PCSK1* are associated with obesity and BMI, while partial LoF variants are not (15). Another study focusing on a specific heterozygous variant (p.Y181H) that was shown in vitro to cause partial PC1/3 deficiency found it was not associated with obesity in a case–control cohort of children and adolescents (72). Those data found no contribution of certain experimentally characterized partial LoF variants in *PCSK1* to adult or childhood obesity risk, which contrasts with our observation of an association between *PCSK1* for both PTVs (predicted complete LoF) and DMG-MISS (predicted partial LoF) with adult BMI. Further studies, ideally integrating computational and experimental approaches, are needed to elucidate the potential effects of partial LoF variants in *PCSK1* on adult or childhood-onset common obesity.

At the variant level, we showed that large population-based cohorts can be a useful resource for screening candidate variants identified in clinical cases. For instance, we assessed the penetrance of 3 *SIM1* heterozygous variants (R171H, R550H, T712I) present in the UKBB, which were first identified in patients with severe early-onset obesity and were functionally characterized as LoF (28). In the UKBB, these variants (with carrier numbers ranging from 8 to 172; Table S2 (59)) showed low obesity penetrance and no significant association with BMI (Table S2 (59)). Similarly, for the *POMC* R236G (73, 74) and Y221C (75) variants, heterozygous carriage were previously reported in index cases of severe early-onset obesity. In the UKBB, we observed a low obesity penetrance for both homozygous (35%, n = 20 total carriers, for R236G; 0%, n = 6 total carriers, for Y211C) and heterozygous carriers (24%, n = 4902 total carriers, for R236G; 23%, n = 2084 total carriers, for Y211C) of these 2 variants. For experimentally characterized variants with sufficient statistical power in the UKBB (defined as those with at least 10 carriers), we found no significant association with either adult BMI or self-reported childhood adiposity. This might be due to the existence of incomplete penetrance (not all carriers of LoF variants show the predicted phenotype), variable expressivity (carriers show differing degrees of phenotypic severity), or the “winner's curse” bias particularly affecting studies of extreme cases with small sample sizes.

Furthermore, our study provided additional evidence for several genes where the haploinsufficiency effect outside of extreme clinical cases remains unclear. On the Genomics England diagnostic gene panel for severe early-onset obesity (V4.0, https://panelapp.genomicsengland.co.uk/), *MC4R*, *PCSK1*, *POMC*, *LEP*, *LEPR*, *SIM1*, and *NTRK2* are classified as “green genes,” indicating high confidence in their impacts on severe early-onset obesity. By testing predicted LoF variants in the population-based UKBB, we recapitulated evidence for heterozygous variant effects in *MC4R*, *PCSK1*, and *POMC.* However, we found no significant associations between heterozygous variants in *LEP*, *LEPR*, *SIM1*, and *NTRK2* with adult BMI or recalled childhood body size. These null results may stem from incomplete penetrance or variable expressivity as mentioned above, or the absence of obesity-associated pathogenic variants in these genes within the cohort. Most of these genes (except *LEP*) are evolutionarily constrained, according to the estimation in gnomAD v2.1.1 (Table S6 (59); pLI > 0.95 or an “observed to expected ratio” upper bound < 0.35 were defined as constrained genes as recommended by Karczewski et al (49)). Therefore, pathogenic variants may be more common in individuals with severe disease who are underrepresented in population studies. Furthermore, *SH2B1* and *KSR2* are currently classified as “amber” genes for severe early-onset obesity due to inconclusive current evidence. Our gene-level burden test using predicted LoF variants did not identify any association with *SH2B1* haploinsufficiency. However, PTVs in *KSR2* were associated with a higher comparative childhood body size, suggesting a potential effect of *KSR2* haploinsufficiency specific to childhood susceptibility to obesity.

Our study has several limitations that should be considered when interpreting the findings. While case-based studies focusing on small numbers of clinically selected individuals may overestimate the penetrance of pathogenic variants, assessment in population-based studies, such as UKBB, are likely to underestimate them. A parallel example is the effect of participant ascertainment on penetrance described for monogenic form of diabetes, where the penetrance of *HNF1A* variants were estimated to range from 32% to 98% depending on case ascertainment (66). Furthermore, it has been shown that individuals with obesity are less likely to participate in population studies, leading to selection bias with healthier than average participants being overrepresented (76, 77). This limitation may particularly apply to *SH2B1*, *NTRK2*, and *SIM1*, as these genes were originally identified in patients with syndromic obesity, characterized by neurobehavioral or developmental abnormalities; such affected carriers would be less likely to participate in the UKBB.

A more robust study design to assess the population prevalence and penetrance of rare variant carriers is a birth cohort, which limits the likelihood of individuals to self-select study entry or drop out due to chronic diseases. A previous birth cohort study reported that the prevalence of heterozygous carriers of in vitro characterized partial or complete LoF variants in *MC4R* is 3 in 1000 (17 carriers in 5724 participants) (21). In the UKBB, the prevalence of predicted LoF (PTV and DMG-MISS) is about 1 in 1000 (554 carriers in 419 581 participants). Notably, these estimates are not directly comparable due to differences in the study design and definitions of LoF variants. Our estimation of LoF was based on bioinformatic predictions, which combine multiple algorithms based on evidence such as sequence conservation and the biochemical properties of amino acid substitutions. Ideally these predictions need to be validated using experimental data to confirm the degree to which the variant may affect protein function and the mechanism of its impact (eg, whether it changes the protein expression level, downstream signaling efficiency, or transcriptional activity). For the DMG-MISS variants, we used the overlap of 2 predictions with a stringent threshold to define deleteriousness, but this comes at the trade-off of a higher false negative rate, potentially leading to an underestimation of the prevalence of pathogenic variant carriage. On the other hand, UKBB has the advantage of a larger sample size, which increases statistical power to detect effects of rare variant carriage. Therefore, findings from multiple approaches should be considered together to provide a comprehensive picture.

Furthermore, restriction to specific protein domains or residues may improve the prediction of variant pathogenicity (78, 79), an approach that was not extensively considered in this study. We examined *NTRK2* as 1 example, as previous studies had identified pathogenic variants specifically within the tyrosine kinase domain. We did not identify an aggregated effect of predicted LoF variants in this domain of *NTRK2* on BMI. Notably, there are many other factors that may affect the pathogenicity of variants. For instance, nonsense variants near to the last exon in the 3′ end of genes are likely to escape nonsense-mediated mRNA decay (80), hence reducing their pathogenicity. Moreover, recent studies have suggested that intrinsic differences in the local constraint of protein domains may affect the pathogenicity of predicted LoF variants (81, 82). This underscores the complexity in variant classifications and the need for further studies to identify factors that determine whether a variant disrupts gene function and confers disease susceptibility. Moreover, we only considered canonical gene transcripts in this study. To fully capture the potential functional consequences of variants in different protein isoforms due to alternative splicing (36, 83), additional transcripts may need to be examined. Finally, our analyses were restricted to individuals of European ancestry, which prevented us from assessing variation in the allele frequency of pathogenic variants across different ancestral groups (84, 85). Consequently, the effect estimates and frequencies of rare variant carriers described here may not generalize to populations of other ancestries.

In summary, this study demonstrated that rare predicted LoF variants in *MC4R*, *PCSK1*, and *POMC* contribute to the population variation in adult BMI. Additionally, LoF variants in these 3 genes, along with *KSR2*, were associated with a higher than average childhood body size. Screening for previously reported functionally characterized variants revealed an absence of such variants for *LEP* (no carriers) and *NTRK2* (1 carrier) in the UKBB, and incomplete penetrance of such variants in the reportedly autosomal dominant genes, including *SH2B1*, *SIM1*, and *KSR2*. Together, these findings emphasize the challenge of determining the population relevance of rare variant effects originally identified in clinically extreme cases. While pedigree-based studies of monogenic or early-onset obesity have been effective in identifying new genes, the increasing availability of sequence data in population cohorts offers a complementary approach for examining allele heterogeneity in genes associated with the common form of disease, and potentially with a wider spectrum of associated phenotypes (86). Furthermore, this study echoed the recent study effort to empathize that the interpretation of penetrance is context-specific and subject to ascertainment bias depending on the study design (65). Finally, this study demonstrated the utility of biobank to potentially assist the variant classification efforts in clinical diagnosis. Consistent with the ACMG and other organizations, we advocate for the use of multiline evidence and more cross-field collaborations to inform variant classifications.

## Data Availability

Original data generated and analyzed during this study are included in this published article or in the data repositories listed in References. Access to the UK Biobank genotype and phenotype data is open to all approved health researchers (http://www.ukbiobank.ac.uk/).

## Abbreviations

BMI: body mass index
DMG-MISS: damaging missense
HC-PTV: high-confidence protein-truncating variant
LoF: loss of function
MAF: minor allele frequency
PGS: polygenic score
PTV: protein-truncating variant
SNP: single nucleotide polymorphism
UKBB: UK Biobank
